# Synchronous Pulmonary Adenocarcinoma and Carcinoid Tumor Diagnosed via a Single-Session Robotic Bronchoscopy: A Case Report

**DOI:** 10.7759/cureus.94044

**Published:** 2025-10-07

**Authors:** Joshua Wortsman, Syed M Naqvi, Dean-Yar Tigrani, Sushant Nanavati, Hasnain Bawaadam

**Affiliations:** 1 Pulmonology, Rosalind Franklin University of Medicine and Science, North Chicago, USA; 2 Pathology, Aurora Medical Center - Kenosha, Kenosha, USA; 3 Interventional Pulmonology, Aurora Medical Center - Kenosha, Kenosha, USA

**Keywords:** adenocarcinoma, carcinoid, pulmonary, robotic bronchoscopy, synchronous multiple primary lung cancers

## Abstract

Synchronous multiple primary lung cancers (SMPLCs) are uncommon, particularly when they consist of histologically distinct tumors, such as adenocarcinoma and carcinoid. Differentiating SMPLCs from intrapulmonary metastases is critical because treatment approaches and prognostic implications vary substantially.

We describe the case of an 82-year-old woman who was found to have both pulmonary adenocarcinoma and a typical carcinoid tumor in separate lobes, diagnosed during a single robotic-assisted bronchoscopy session. This case underscores the importance of considering SMPLCs in patients with multifocal pulmonary lesions, highlights the diagnostic utility of advanced bronchoscopic platforms that enable sampling of multiple sites in a single procedure, and points to emerging molecular evidence suggesting that adenocarcinoma and carcinoid tumors may share a common progenitor cell origin.

## Introduction

Lung cancer is the leading cause of cancer-related mortality worldwide and in the United States of America [1]. Although pulmonary metastases from extrathoracic malignancies are common, the occurrence of synchronous multiple primary lung cancers (SMPLCs) remains rare, with an incidence of less than 2% of lung cancer cases [2]. By definition, SMPLCs consist of two or more primary tumors arising simultaneously but independently. They may share histology or, less commonly, display discordant histology [3].

Accurate classification of SMPLCs is critical, as management strategies depend on the underlying tumor type [4]. Advances in technology, such as navigational and robotic bronchoscopy for precise sampling, and next-generation sequencing (NGS) to guide targeted therapies, have enhanced both diagnostic accuracy and treatment outcomes. These innovations underscore the importance of obtaining tissue from multiple lesions when separate primaries are suspected. In this report, we present an unusual case of synchronous pulmonary adenocarcinoma and carcinoid tumor, both diagnosed during a single-session robotic bronchoscopy.

## Case presentation

**Figure 1:**
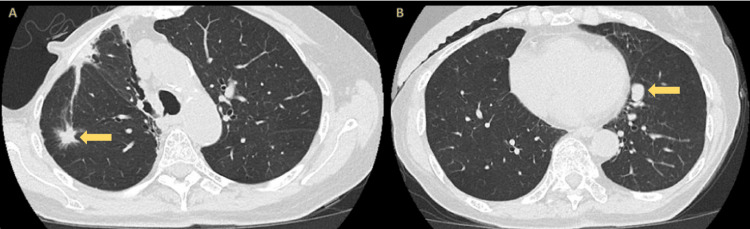
CT imaging demonstrating two suspicious pulmonary lesions Panel A: CT showing a spiculated lateral subpleural right upper lobe nodule measuring 11 × 12 mm (arrow), confirmed as moderately differentiated adenocarcinoma. Panel B: CT showing a juxta-cardiac left lower lobe nodule measuring 10 × 16 mm (arrow), confirmed as a typical carcinoid tumor. CT, computed tomography

**Figure 2:**
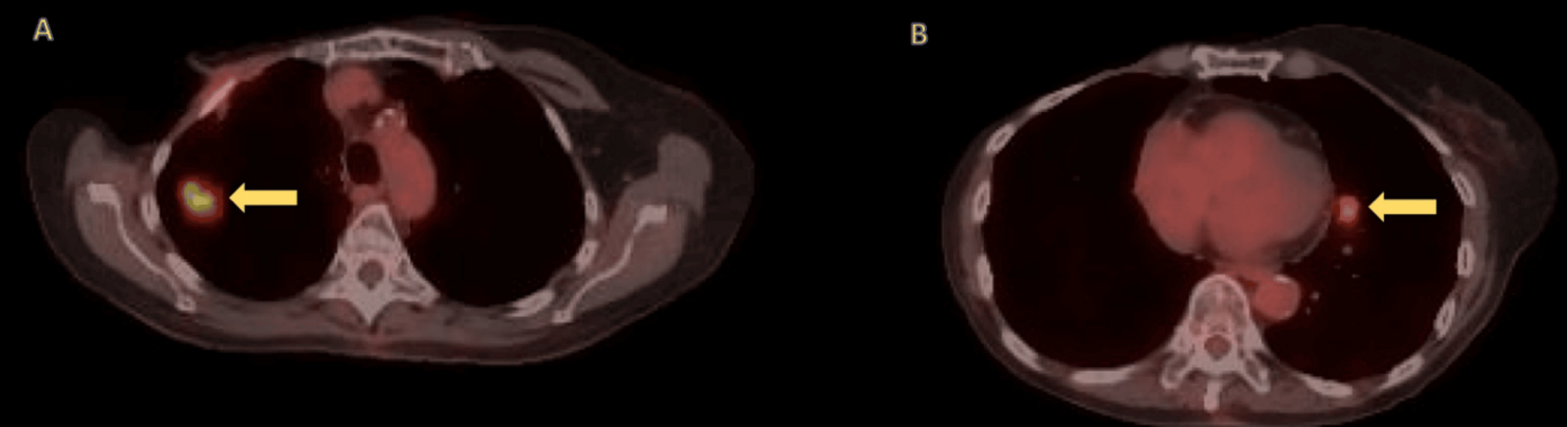
PET/CT imaging demonstrating two FDG-avid pulmonary lesions Panel A: Axial PET/CT showing a spiculated lateral subpleural right upper lobe nodule measuring 11 × 12 mm with intense FDG uptake (SUV max 8.2) (arrow), confirmed as moderately differentiated adenocarcinoma. Panel B: Axial PET/CT showing a juxta-cardiac left lower lobe nodule measuring 10 × 16 mm with moderate FDG uptake (SUV max 3.5) (arrow), confirmed as a typical carcinoid tumor. PET/CT, positron emission tomography/computed tomography; FDG, fluorodeoxyglucose; SUV, standard uptake value

An 82-year-old woman with a history of estrogen receptor-positive, progesterone receptor-positive, HER2-negative right breast cancer, treated with mastectomy and radiation in 1979, experienced recurrences in 1985 and 2020. In early 2025, surveillance chest computed tomography (CT) revealed a progressive, spiculated right upper lobe (RUL) nodule measuring 11 × 12 mm, and a new juxtacardiac left lower lobe (LLL) lesion measuring 10 × 16 mm, as shown in Figure 1. Positron emission tomography (PET)/CT demonstrated intense fluorodeoxyglucose (FDG) uptake in the RUL lesion (standard uptake value (SUV) max 8.2) and moderate uptake in the LLL lesion (SUV max 3.5), as shown in Figure 2, without evidence of mediastinal or distant metastasis.

**Figure 3:**
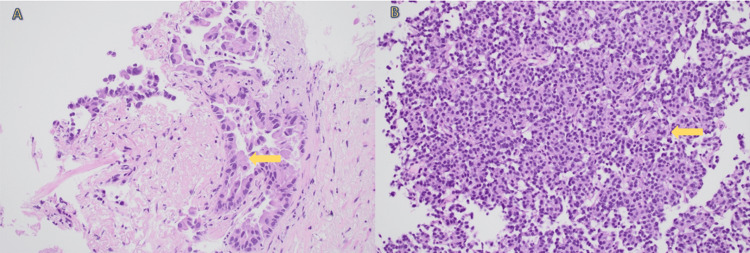
Histopathology slides Hematoxylin and eosin (H&E) stained sections of lung biopsy specimens. Panel A: Right upper lobe lesion demonstrating adenocarcinoma (400×, arrow). Panel B: Left lower lobe specimen showing carcinoid tumor (400×, arrow).

Given the presence of two morphologically distinct nodules, she underwent single-session robotic-assisted bronchoscopy. The RUL lesion was sampled with fine-needle aspiration (FNA), transbronchial forceps biopsy, and cryobiopsy, while the LLL lesion was sampled with FNA and forceps biopsy. Histopathology revealed moderately differentiated adenocarcinoma in the RUL specimen and a typical carcinoid tumor in the LLL specimen, as shown in Figure 3, with no evidence of mediastinal nodal involvement. These findings established the diagnosis of SMPLCs rather than metastatic breast carcinoma, specifically identifying distinct primary tumors: adenocarcinoma and typical carcinoid. Following multidisciplinary evaluation and consideration of the patient’s age and comorbidities, the decision was made to proceed with stereotactic body radiation therapy (SBRT) targeting both tumor lesions, with subsequent surveillance imaging.

## Discussion

SMPLCs pose a significant diagnostic challenge due to their rarity and frequent mimicry of intrapulmonary metastases. While incidence estimates vary, synchronous presentations are considerably less common than metachronous cases, which are subsequent tumors that arise six months to years after an initial primary tumor [5]. Risk factors, such as tobacco exposure and environmental carcinogens, are implicated in their pathogenesis, consistent with the concept of field cancerization, whereby widespread epithelial injury predisposes to multifocal tumorigenesis [6].

The coexistence of adenocarcinoma and carcinoid tumor is especially rare, as most SMPLCs involve histologically similar neoplasms, such as multiple adenocarcinomas or squamous cell carcinomas [7]. Carcinoid tumors constitute only a small subset of lung cancers, and their synchronous presentation with adenocarcinoma has been described only in isolated reports [3]. When present together, these tumors may manifest as: (a) synchronous primaries arising in separate lung fields; (b) collision tumors within a single lesion that retain distinct histologic borders and arise independently; or (c) composite tumors, thought to originate from a shared progenitor cell, and often exhibiting overlapping mutational profiles [8].

The underlying biology of adenocarcinoma-carcinoid coexistence remains incompletely understood. Collision tumors suggest independent origins, whereas composite lesions likely represent divergent differentiation of a common epithelial progenitor. Increasing molecular evidence supports the composite lesions hypothesis: shared alterations, such as KRAS, NF1, and BRAF V600E mutations, have been reported in both adenocarcinoma and carcinoid components, suggesting a monoclonal origin in select cases [8,9]. These findings underscore the importance of NGS in refining tumor classification and guiding therapy.

From a diagnostic perspective, distinguishing SMPLCs from intrapulmonary metastasis is often difficult. Multiple pulmonary nodules are common, and although radiographic features such as spiculation, lobulation, or vascular convergence may suggest malignancy [10], imaging alone cannot reliably distinguish separate primaries from metastasis. Histologic confirmation is, therefore, essential. In the present case, robotic-assisted bronchoscopy enabled minimally invasive sampling of anatomically distinct nodules in a single session, facilitating rapid diagnosis while avoiding multiple invasive procedures. Robotic platforms have demonstrated superior navigation accuracy and diagnostic yield in small or peripheral lesions, making them particularly valuable in the evaluation of multifocal disease [11].

Management of SMPLCs requires staging and individualized treatment for each tumor, consistent with current TNM (Tumor, Node, Metastasis) guidelines [4]. Treatment strategies diverge significantly between adenocarcinoma and carcinoid tumors: adenocarcinoma management is stage- and mutation-dependent, incorporating surgery, systemic chemotherapy, targeted therapy, or immunotherapy; whereas typical carcinoids are indolent neuroendocrine tumors for which surgical resection remains the only curative option. Prognosis is favorable for typical carcinoids, with five-year survival rates of 90%-100%, compared to 60%-80% for atypical carcinoids [12]. In our patient’s case, given her advanced age and comorbidities, definitive local therapy with SBRT to both lesions was deemed the most appropriate management strategy. In scenarios of SMPLCs with discordant histologies, multidisciplinary deliberation is critical to tailoring treatment plans that account for the distinct biological behaviors, growth kinetics, and therapeutic sensitivities of each tumor type.

## Conclusions

This case reinforces several important clinical considerations. First, SMPLCs should be considered in the differential diagnosis for patients presenting with multifocal pulmonary nodules, even in those with a history of extrathoracic malignancy. Second, advanced bronchoscopic techniques, such as robotic-assisted bronchoscopy, provide an efficient and minimally invasive diagnostic approach that allows simultaneous sampling of multiple lesions. Finally, ongoing molecular studies suggest that adenocarcinoma-carcinoid tumors may not merely represent coincidental lesions but could share a common progenitor, underscoring the need for further genomic research.
